# Long-Read Sequencing as a Diagnostic Tool for Primary Ciliary Dyskinesia

**DOI:** 10.1155/crig/5109434

**Published:** 2025-11-26

**Authors:** Liora H. Feshbach, Morgan Similuk, Laura M. Amendola, Katie L. Lewis, Magdalena A. Walkiewicz, Mari Tokita, Rajarshi Ghosh, Andrew Lipton

**Affiliations:** ^1^Centralized Sequencing Program, National Institute of Allergy and Infectious Diseases (NIAID), NIH, Bethesda, Maryland, USA; ^2^National Heart, Lung, and Blood Institute (NHLBI), NIH, Bethesda, Maryland, USA

## Abstract

Primary ciliary dyskinesia (PCD) is a rare, inherited disease resulting from abnormal structure and/or function of cilia. To date, pathogenic variants in over 50 genes have been reported as causes of PCD. One of the genes, *HYDIN*, presents a diagnostic challenge due to the existence of *HYDIN2*, a highly homologous pseudogene that significantly complicates accurate molecular diagnosis. Here, we present a 43-year-old female with a clinical diagnosis of PCD seeking molecular diagnosis underlying her disease. Short-read genome sequencing detected two potentially pathogenic *HYDIN* variants (c.5416C > T and c.3786-1G > T), but clinical validation was hindered due to the pseudogene overlap. Using clinical long-read genome sequencing (lrGS), we confirmed the presence of both *HYDIN* pathogenic variants and a *trans* configuration, establishing the molecular diagnosis for this patient. This case highlights the promise of lrGS in diagnosing *HYDIN*-related PCD and demonstrates that offering lrGS to PCD patients, especially those with suspected *HYDIN* variants, could enhance diagnostics, disease management, and genetic counseling.

## 1. Introduction

Primary ciliary dyskinesia (PCD) is a rare, genetically heterogenous disorder characterized by abnormal cilia, leading to a build-up of mucus in the respiratory tract, chronic oto-sino-pulmonary disease, and progressive, irreversible lung damage. Clinically, PCD presents similarly to other chronic respiratory disorders, such as cystic fibrosis (CF) and non-CF bronchiectasis, resulting in overlapping management strategies. Symptoms typically manifest in infancy and include neonatal respiratory distress, recurrent otitis media with effusion, chronic cough, nasal congestion and recurrent lower respiratory infections [1]. Over time, chronic oto-sino-pulmonary disease leads to bronchiectasis in most adults with PCD, causing permanent airway dilation and further predisposing them to recurrent infections. Additionally, fertility is reduced in both sexes, with nearly 100% of males having abnormal sperm motility and infertility. Laterality defects are also common in PCD. Approximately 50% of PCD patients have situs inversus totalis and a smaller fraction have more subtle differences such as intestinal malrotation or polysplenia [1].

The number of known PCD-associated genes has more than doubled over the last decade with pathogenic variants in over 50 genes currently known to cause PCD [2] PCD is predominantly inherited in an autosomal recessive manner, with exceptions including *FOXJ1* (autosomal dominant), *PIH1D3*, and *OFD1* (X-linked) [3]. While all forms of PCD share core clinical characteristics, genotype–phenotype correlations exist and are largely governed by the impacted cilia structures. For example, individuals with PCD caused by defects in *RSPH3* and *HYDIN* (among others) typically do not exhibit laterality defects, as these genes are involved in the formation and function of cilia within the respiratory and reproductive systems but not in the specialized embryonic nodal cilia that are crucial for establishing left–right asymmetry during embryonic development [4]. *HYDIN* is one of the most common PCD-causing genes, accounting for 7%–9% of all PCD cases in some populations [5]. One potentially distinguishing feature of PCD caused by *HYDIN* defects is the possible association with connective tissue impairments, which may lead to joint hypermobility and scoliosis; this observation stands to be replicated in larger cohorts [5].

Significant advances have been made in PCD diagnostics, including ciliary motility studies, nasal nitric oxide measurements, and genetic testing. However, these approaches have limitations, contributing to persistent challenges in diagnosis [1]. While genetic testing for individuals with suspected PCD can identify a molecular etiology and inform recurrence risk counseling, up to a third of patients with a clinical diagnosis of PCD remain without a molecular diagnosis despite extensive genetic testing [6]. Molecular testing approaches for PCD include single gene testing, targeted gene-panel testing, and genomic sequencing tests, including exome and genome sequencing. However, pseudogene interference complicates variant detection, particularly for *HYDIN*, which has a pseudogene on another chromosome, *HYDIN2*, that shares 98% sequence identity with *HYDIN*. In recent years, emerging long-read genome sequencing (lrGS) technologies have improved diagnostic rates for rare diseases by overcoming limitations inherent to short-read genome sequencing (srGS) [7, 8]. Deletions, insertions, and rearrangements are challenging to identify using srGS, while lrGS can generate reads from 1000 to 1 million base pairs in length, making it easier to identify these variant types. Similarly, lrGS allows for more accurate resolution of highly homologous regions, making it particularly valuable for genes affected by pseudogene interference. lrGS can also uncover DNA methylation patterns [8].

We present a patient with a clinical diagnosis of PCD who underwent both srGS and lrGS, demonstrating how advanced genomic technologies can resolve challenging molecular diagnostic cases. The findings underscore the importance of integrating lrGS into diagnostic workflows to reduce time to diagnosis, enhance diagnostic yield, and improve patient access to precise prognostic and therapeutic guidance.

## 2. Case Description

### 2.1. Clinical History

A 43-year-old female presented to the National Institute of Health (NIH) Clinical Center for clinical research evaluations of long-standing pulmonary disease. She first experienced respiratory distress at birth. In childhood, she developed recurrent infections including sinusitis, pneumonias, and otitis media, requiring ear tube placement.

Sweat chloride testing by quantitative pilocarpine iontophoresis was not consistent with a diagnosis of CF. Endobronchial biopsy was performed at age 6, but transmission electron microscopy results are unavailable. She went on to be clinically diagnosed with PCD. Airway clearance management was initiated, including use of a percussion vest before bed, use of Acapella (a positive expiratory pressure oscillatory device) when traveling, huff coughs as needed, and 7% saline via nebulizer before bed.

As a teenager, the patient had chronic sinusitis which was treated surgically via the placement of a sinus stent. At age 24, she was hospitalized for pneumonia with a parapneumonic effusion, requiring chest tube drainage. Subsequently, she developed worsening pulmonary symptoms and hemoptysis. The hemoptysis was not improved with cessation of her hypertonic saline washes but resolved upon a bilateral bronchial artery embolization. She had normal immunoglobulin levels and no history of impaired immunoglobulin production, autoimmunity, or lymphoproliferation. She had multiple left and right shoulder dislocations and was found to have joint hypermobility and scoliosis.

Recent pulmonary function tests showed a low-normal forced vital capacity (FVC) of 79% of predicted and a forced expiratory volume in one second (FEV1) of 59% of predicted (*z*-score 3.16), resulting in a FEV1/FVC ratio of 0.61 (*z*-score −3.05), which is below the lower limit of normal. In addition to the below average spirometry, her residual volume to total lung capacity (RV/TLC) ratio of 43% indicates air trapping occurs after full exhalation. These findings are consistent with the obstructive pulmonary complications seen in PCD.

Nasal nitric oxide (nNO) levels in exhalation against resistance can be used in the evaluation for PCD. For individuals with PCD, dysfunctional cilia in the nasal epithelium may affect the production and/or clearance of NO; however, the specific physiology is unknown. On her most recent evaluation, this patient's nNO was 26 nL/min. Scores below 77 nL/min are suggestive of PCD, thus the patient's score is consistent with her clinical diagnosis of PCD.

**Figure 1:**
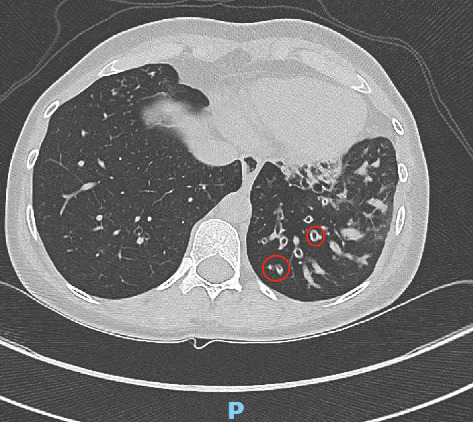
This image shows more severe bronchiectasis seen in the lower lung fields in this patient. Bronchiectasis is more severe in the lower lungs than the upper, as is typical in PCD as compared with CF, where the upper lobes are usually more severely affected. Bronchiectasis is noted radiographically when the diameter of the bronchus exceeds the diameter of the accompanying bronchial artery, as in the two pairs with red circles.

Finally, this subject has a history of suppurative airway disease with repeated demonstration of neutrophils. However, she has only had one isolate to date of *Pseudomonas aeruginosa* and *Staphylococcus aureus*. A CT scan confirms no laterality defects (Figure 1).

**Figure 2:**
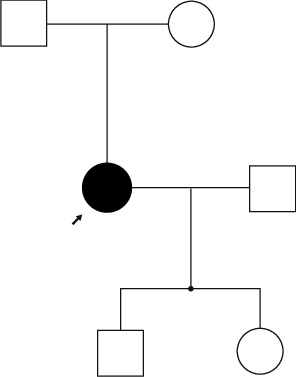
Pedigree of our patient. She is an only child and is the only one affected in this pedigree. Neither her parents nor her children are affected.

The family history is grossly negative for respiratory disorders (Figure 2). The participant has a son and daughter, both of whom are healthy. She does not report using any fertility assistance. Her son has a diagnosis of Hirschsprung's disease but no history of respiratory issues. Neither the participant's father nor her mother has a history of PCD.

### 2.2. Genetic Testing

Genome sequencing was performed by the NIH Centralized Sequencing Program (CSP) [9]. The CSP provides clinical srGS to patients enrolled on research protocols at the NIH Clinical Center with the overall goal of identifying genetic contributions to disease and being a genomic resource for human subject's research studies at the NIH.

This participant's srGS included analysis and return of results for primary and secondary findings. Secondary findings are defined as variants in genes associated with rare, serious disorders for which evidence-based medical management exists. Genome analysis and variant prioritization were performed in accordance with American College of Medical Genetics/Association of Molecular Pathologists (ACMG/AMP) recommendations, integrating population frequency, predicted functional impact, inheritance models, published literature evidence, and phenotypic correlation. Following sequencing, variants within coding and noncoding regions were annotated using Human Genetic Variation Society nomenclature, population allele frequency, presence in clinical databases such as ClinVar and Human Genetic Mutation Database, in silico variant deleteriousness predictors, gene–disease association in Online Mendelian Inheritance in Man, and genotyping quality. We systematically filtered against gnomAD v4 with a primary threshold of minor allele frequency < 0.01, while allowing for exceptions in cases of known pathogenic alleles exceeding this frequency or variants for which literature evidence existed at the time of analysis. For genes associated with recessive disorders, special attention was given to scenarios where only a single pathogenic/likely pathogenic variant was identified in a gene with compelling phenotypic overlap; in these cases, coverage metrics, structural variant (SV) analysis, and review of common risk alleles were performed to evaluate for a potential second pathogenic allele. We additionally perform review of structural variant called by Genome Analysis Tool Kit-SV from the srGS data in accordance with ACMG/AMP recommendations. This strategy ensured systematic prioritization of variants most likely to contribute to disease for both primary diagnostic findings and ones that are medically actionable (secondary findings per ACMG/AMP recommendations).

**Figure 3:**
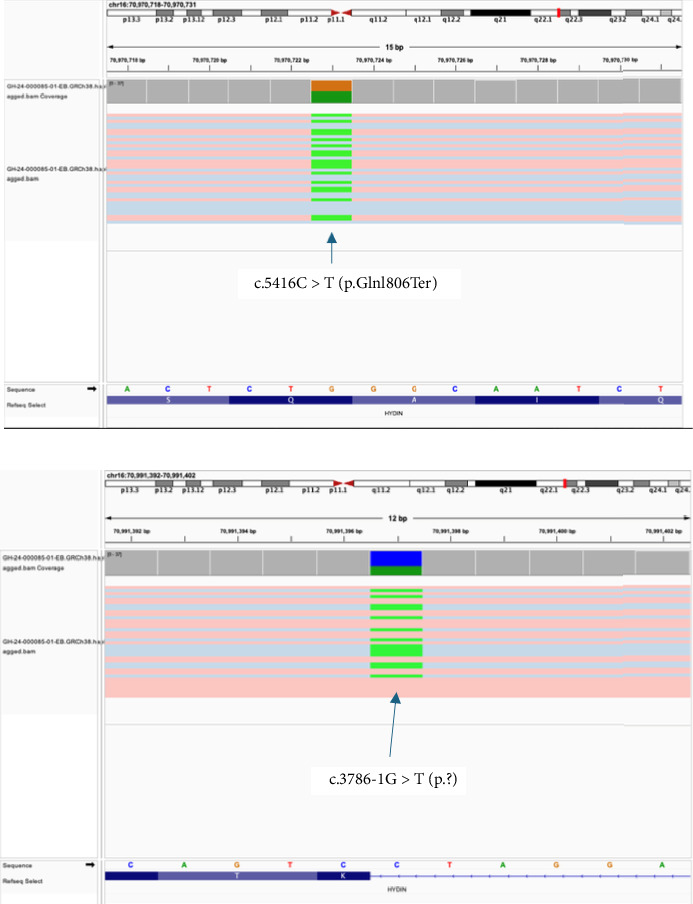
Long-read sequencing visualization and variant locations within the *HYDIN* gene. A heterozygous c.5416C > T (p.Glnl806Ter) nonsense variant [chrl6:70970723 (GRCh38) NM_001270974.2] and a heterozygous c.3786-1G > T (p.?) splice acceptor variant [chr16:70991397 (CRCh38) NM_001270974.2]. These variants were present on opposite chromosomes (*trans* configuration) providing a molecular diagnosis for this participant's PCD. The blue and red (haplotypes) are in *trans*.

SrGS analyzed rare variants in all known PCD*-*associated genes (Supporting Table 1). SrGS detected two variants of interest in *HYDIN*: a heterozygous c.5416C > T (p.Glnl806Ter) nonsense variant [chrl6:70970723 (GRCh38) NM_001270974.2] and a heterozygous c.3786-1G > T (p.?) splice acceptor variant [chr16:70991397 (CRCh38) NM_001270974.2]. While these variants were highly suspicious for a molecular diagnosis of *HYDIN*-related PCD, their exact genomic location could not be confirmed due to technical limitations of srGS. Specifically, the presence of a highly homologous pseudogene, *HYDIN2*, which shares 98% sequence identity with *HYDIN*, made it impossible to distinguish whether these variants resided in *HYDIN* or its pseudogene. To resolve this ambiguity, lrGS was performed. lrGS not only confirmed that these variants were present in *HYDIN*, and not *HYDIN2*, but also established that these variants were present on the opposite chromosomes (*trans* configuration) providing a molecular diagnosis for this participant's PCD (Figure 3). With their location in HYDIN confirmed, we were able to classify the variants as pathogenic for PCD.

SrGS also detected a likely pathogenic missense variant in the *TNFRSF13B* gene [chr17:16948873 (GRCh38) NM_012452.3, c.310T > C (p.Cys104Arg)]. Pathogenic variants in *TNFRSF13B* are associated with common variable immunodeficiency (CVID) [OMIM# 240500], thus this variant was reported as a primary finding based on possible clinical overlap with the participant's history of recurrent infection. As part of our standard genomic analysis, we reviewed all variants present at < 1% minor allele frequency in gnomAD in genes that are associated with disease with OMIM; a molecular diagnosis or compelling variants of uncertain significance in a gene associated with connective tissue disorders was not identified. Despite the patient's history of hypermobility and recurrent dislocations, we did not conclusively find any gene variants related to connective tissue disease.

## 3. Discussion

We report a case in which lrGS provided a definitive molecular diagnosis for a patient with a longstanding clinical history of PCD. This case contributes to the growing body of evidence supporting the clinical utility of lrGS, particularly in resolving genetic variants that are confounded by pseudogenes. While srGS remains a powerful tool for detecting many diagnostic variants in rare diseases, its limitations include the inability to accurately map sequences in highly homologous regions, difficulty in resolving complex structural variants, and challenges in detecting epigenetic modifications such as DNA methylation [10, 11] that can be overcome by lrGS.

Consistent with previous studies on the application of lrGS in rare disease diagnostics [12], this case demonstrates the ability of advanced sequencing technologies to resolve previously undiagnosed cases. In this case, lrGS was essential in determining whether the patient's potentially diagnostic variants in *HYDIN* resided in the disease-associated *HYDIN* or its nearly identical pseudogene, *HYDIN2,* which is not associated with disease. Additionally, it allowed molecular geneticists to establish the phase of these two variants, confirming her molecular diagnosis. Access to lrGS is especially valuable for patients whose parents are not available for testing.

Pathogenic variants in *HYDIN* are typically associated with a specific PCD phenotype [5], which in most cases is characterized by normal organ laterality, low nNO, and an increased risk of bronchiectasis. This participant's molecular diagnosis of *HYDIN-*related PCD is consistent with her clinical history. It is estimated that many PCD patients with pathogenic variants in *HYDIN* are molecularly underdiagnosed because variation identified via the most common forms of genetic testing cannot be differentiated from variation in the associated pseudogene, *HYDIN2* [5].

In addition to the *HYDIN* variants, this patient was also found to have a likely pathogenic variant in *TNFRSF13B*, a gene associated with CVID. While CVID is typically characterized by impaired immunoglobulin production, its penetrance is incomplete [13]. Given her clinical history, it is unlikely that this variant is the primary driver of her recurrent infections.

This result enables accurate reproductive risk counseling, as the inheritance pattern and recurrence risks can now be determined. Additionally, as precision therapies for *HYDIN*-related PCD emerge, this molecular information may facilitate future access to personalized treatments. Beyond medical implications, the psychosocial impact of a confirmed diagnosis is significant, providing closure after a prolonged diagnostic odyssey and offering valuable information to the patient and her family.

This case is fundamentally concordant with another study investigating lrGS of *HYDIN* variants of 437 families [14]. Varying approaches, including innovative analytical methods with Short-Read Next Generation Sequencing with bioinformatic masking of *HYDIN2*, could be considered as an alternative to lrGS for PCD patients.

This case report has limitations. The findings are based on a single individual, which limits their generalizability to broader PCD populations. While lrGS was instrumental in achieving a molecular diagnosis, the case does not compare this approach directly to alternative diagnostic methods across a broader group and thus does not inform how frequently lrGS is required for diagnosis of *HYDIN*-related PCD. Additionally, cost-effectiveness and feasibility of implementing lrGS in routine diagnostic workflows for PCD are beyond the scope of this case report and remain to be established. Lastly, while these variants are likely to be pathogenic based on one leading to a stop codon/nonsense mutation and the other one leading to a splice variant, RT-PCR and some additional functional studies of cilia would help to confirm the lack of normal *HYDIN* transcripts and ciliary function.

Despite these limitations, this case highlights the transformative potential of lrGS to resolve challenging molecular diagnoses and underscores the importance of integrating advanced genomic technologies into clinical practice.

## 4. Conclusions

LrGS enabled a definitive molecular diagnosis of *HYDIN-*related PCD in this patient, overcoming limitations of conventional methods. This case underscores the importance of integrating advanced genomic technologies to improve diagnostic accuracy for rare disease diagnostics. As lrGS becomes more accessible, its broader adoption could transform rare disease diagnostics and precision medicine.

## Supporting Information

Supporting Table 1: This gene list was referenced during data analysis. The first column reflects the ensemble gene ID, and the second column represents the commonly used gene name.
